# Modelling the differential effects of age on transcranial magnetic stimulation induced electric fields

**DOI:** 10.1088/1741-2552/ac9a76

**Published:** 2023-03-16

**Authors:** Mansour Alawi, Poh Foong Lee, Zhi-De Deng, Yong Kheng Goh, Paul E Croarkin

**Affiliations:** 1 Lee Kong Chian Faculty of Engineering & Science, University Tunku Abdul Rahman, Kajang, Malaysia; 2 Noninvasive Neuromodulation Unit, National Institute of Mental Health, NIH, Bethesda, MD, United States of America; 3 Department of Psychiatry and Psychology, Mayo Clinic, Minnesota, MN, United States of America

**Keywords:** anatomical variabilities, computational simulation, electric field modeling, noninvasive brain stimulation, transcranial magnetic stimulation

## Abstract

**Objective.:**

The therapeutic application of noninvasive brain stimulation modalities such as transcranial magnetic stimulation (TMS) has expanded in terms of indications and patient populations. Often neurodevelopmental and neurodegenerative changes are not considered in research studies and clinical applications. This study sought to examine TMS dosing across time points in the life cycle.

**Approach.:**

TMS induced electric fields with a figure-of-eight coil was simulated at left dorsolateral prefrontal cortex regions and taken in vertex as a control region. Realistic magnetic resonance imaging-based head models (*N* = 48) were concurrently examined in a cross-sectional study of three different age groups (children, adults, and elderlies).

**Main results.:**

Age had a negative correlation with electric field peaks in white matter, grey matter and cerebrospinal fluid (*P <* 0.001). Notably, the electric field map in children displayed the widest cortical surface spread of TMS induced electric fields.

**Significance.:**

Age-related anatomical geometry beneath the coil stimulation site had a significant impact on the TMS induced electric fields for different age groups. Safety considerations for TMS applications and protocols in children are warranted based on the present electric field findings.

## 1. Introduction

Transcranial magnetic stimulation (TMS) modulates the brain’s cortical activities through the induction of intracranial electric fields [1] for therapeutic purposes and neurophysiological studies across a wide age range [2, 3]. Numerous studies demonstrate the utility of TMS as a diagnostic tool for neurophysiological abnormalities and motor mapping in children [4]. Therapeutic applications of TMS in children have developed slowly because of safety concerns and the practical aspects of study design and execution [5, 6]. For young adults, a study reported the safety and efficacy of TMS in reducing the symptom of this group with attention-deficit/hyperactivity disorder [7]. Another recent study provided preliminary evidence for high-frequency repetitive TMS treatments in the treatment of adolescents with suicidal ideation and depressive symptoms [8]. On the other side of the life cycle, TMS has been used in geriatric patients for cortical mapping [9], treatment of neurocognitive disorders [10], and the treatment of major depressive disorder [11]. However, the nuances of age-related changes in the brain during neurodevelopment, cortical atrophy, and other age-related anatomical variation for designing TMS therapeutic protocols is still not fully understood or appropriately considered in research design [12].

Prior experimental studies have considered the impact of age on TMS effects. A recent report suggested that those age-related changes impact late synaptic inputs to corticospinal neurons thereby influencing fine motor performance [13]. Comparison of the degeneration within the central nervous system due to age-related weakness was also found in the corticospinal output in the primary motor cortex between young adults and the elderly [14]. Interestingly, intracortical inhibition among young and older adults showed no age-related differences with conventional TMS coils [15]. Another experimental study comparing children and adults reported increased corticospinal inhibition in children that decreased with age [16]. A single-pulse TMS protocol is usually used to map the motor function of the cerebral cortex, where a TMS single pulse is delivered to the motor cortex to elicit a motor evoked potential (MEP) in the corresponding muscles. In some studies, implementing this protocol with children was problematic because of the low reliability resulting from failure to measure motor threshold in children, which was attributed to higher motor thresholds and anatomical differences [17]. A prior study investigated the differences in MEP measurements between healthy children, adolescents, and adults reported the phase of the oscillatory response to TMS were less consistent with age [9]. These findings demonstrate the need for research comparing the anatomical and physiological impact of age on TMS cortical stimulation. Prior research demonstrated that decreases in brain size yields increased values of all TMS induced fields [18]. However, the study did not include the head model of middle-aged adults and the elderly. There is an increase in experimental studies across age groups to address the knowledge gaps related to age. Computational field modeling is an important tool for examining these questions related to age effects of TMS stimulation [19].

Modeling-induced TMS fields for age-related anatomical changes in the developing and aging brains could help optimize the safety, feasibility, precision, and efficiency of TMS use in children and elderly patients [20, 21]. The recent improvements on the computational modeling using finite element method (FEM) and individualized, magnetic resonance imaging (MRI)-derived head models have provided unique insights on the TMS parameters and the inter-individual variability in induced intracranial electric fields strengths and distribution [22]. A previous TMS study that used FEM quantified induced electric fields in healthy adults and revealed the cortical regions that experienced the highest electric fields strength following the TMS intervention [6, 23]. A prior study found that the FEM simulations of the electric field strength, including gyral folding patterns and tissue conductivity anisotropy, increased the targeting accuracy of TMS in the mapping or modulation of human brain circuits [14]. Studies have used FEM to optimize dose and coil placement in therapeutic TMS for stroke rehabilitation in children [15].

This study sought to examine the TMS induced electrical field in the left dorsolateral prefrontal cortex (L-DLPFC) and vertex as a control region-specific in children (4–12 years old), adults (21–40 years old), and elderly (75–84 years old). We expected that these three discrete age groups would have variability in anatomy. The vertex region served as an active control, consistent with prior TMS studies [24]. The effects of the induced electric field in the region of L-DLPFC is of interest since it is frequently a therapeutic target in TMS protocols [25], in addition, the motor region was modeled and compared for this parameter. We hypothesized that age-related variation of anatomical factors would impact electric field modeling.

## 2. Materials and methods

### 2.1. Dataset processing and sample grouping

MRI datasets were obtained from OpenNEURO (https://openneuro.org/) an open platform database for MRI images from different functional MRI studies [26, 27]. The datasets were acquired using scanners with field strength of 3 T (Siemens Tim Trio) and included high resolution T1-weighted MRI scans for a total of 403 subjects with an age range between 3 and 84 years of age. For this modeling study, 60 healthy subjects were randomly selected from the datasets using the random selection library in Python 3 (Python Software Foundation, www.python.org/), the number of subjects were chosen to examine putative differences in age effects on TMS between the age groups and to get aligned with other similar studies’ sample size [21, 28]. A prior power analysis was conducted using G^∗^*Power* statistical analysis program for sample size estimation, which suggested *n* = 66 for effect size of 0.40 (significant criterion of *α*= 0.05 and power = 0.82) for one-way analyses of variance (ANOVA) in this study.

The finalized sample size of 60 subjects had a balanced distribution of male and female subjects in three age categories: children (*n* = 20, 4–12 years old), adults (*n* = 20, 22–37 years old), and elderly (*n* = 20, 75–84 years old). The quality of the selected MRI scans was further assessed using the image quality report generated by the SPM12’s Computational Anatomy Toolbox (CAT12) during the segmentation process. These three categories were selected to address the effects of the neurodevelopmental differences in children [6, 29], to examine age-related changes in elderly patients [30, 31], and to compare both groups to the relatively stable neurological anatomy adult groups [6, 29]. The dataset-related links and selection samples as head model details are shown in the Supplementary File.

### 2.2. Tissue segmentation and TMS induced fields calculation

**Figure 1.**
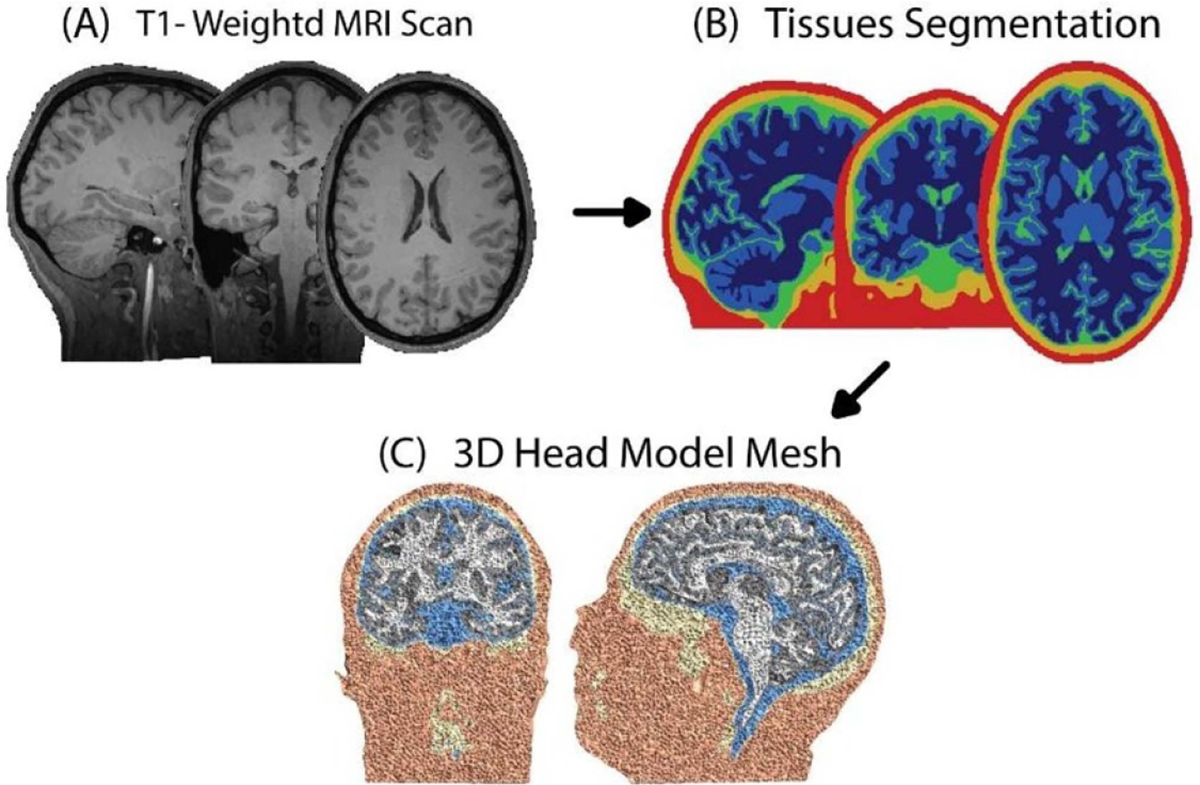
Tissues segmentation and 3D head model generation. (A) Acquisition of T1-weighted MRI anatomical scans. (B) Segmentation of MRI scan into five tissue types. (C) Calculation of the head volume mesh using tetrahedrons elements.

The subject head model segmentation and TMS induced field simulations were performed by employing SimNIBS pipeline (version 2.1.2) [32, 33]. For each subject, the process started with tissue segmentation which began with standardizing the anatomical MRI scan orientation to the radiological left-anterior-superior coordination with a Voxel size of [1 1 1] mm to eliminate differences in the scanning settings and facilitate data comparison among the groups. This was followed by operating the SimNIBS *headreco* function. The T1-weighted MRI scan was segmented into five tissue types: scalp, skull, cerebral spinal fluid (CSF), grey matter (GM) and white matter (WM). This step was performed via MATLAB (Mathworks, MA) and the segmentation routines from statistical parametric mapping (SPM12) [34] and the SPM12’s CAT12 toolbox. The segmentation masks were checked slice-by-slice to ensure accurate tissue segmentation by using Freeview [35]. Subsequently, the head model tetrahedral mesh was generated based on the segmented masks using Gmsh [36]. Following these steps, the three-dimensional head model mesh was fully constructed to be used in TMS induced fields’ simulation (figure 1).

The TMS induced electric fields was simulated with SimNIBS functions by applying FEM to solve the equation that governed the TMS induced electric fields in the head meshes [32, 37]. The simulation coil current rate of change was set at 1 Ampere/micro-second (A*μ*s^−1^) for the purpose of enlarging the TMS induced electric field values and showing the effects at 50% of the stimulator output, this was acceptable because the coil current rate of change does not affect the statistical differences within or between the age groups. The conductivity values for segmented tissue types from previously established studies were used during TMS fields FEM calculation (table 1) [38, 39]. The TMS coil used in this simulation was the SimNIBS software’s Magstim 70 mm figure-of-eight coil with monophasic pulse, the coil is represented by two circular disk of 5 cm radius, each disk is consisting of ten rings representing the coil wires, each of the ring is divided into set of elements that represent the coil dipoles [39]. Using the stated dipoles architecture for the coil electric field calculation based on Faraday’s law was reported to produces a sufficiently accurate representation for the electromagnetic fields induced in the real coil and do not severely suffer from eddy-current effects in the coil architecture that consider the current flow uniformly through the coil’s wires [40].

### 2.3. Coil placement

**Figure 2.**
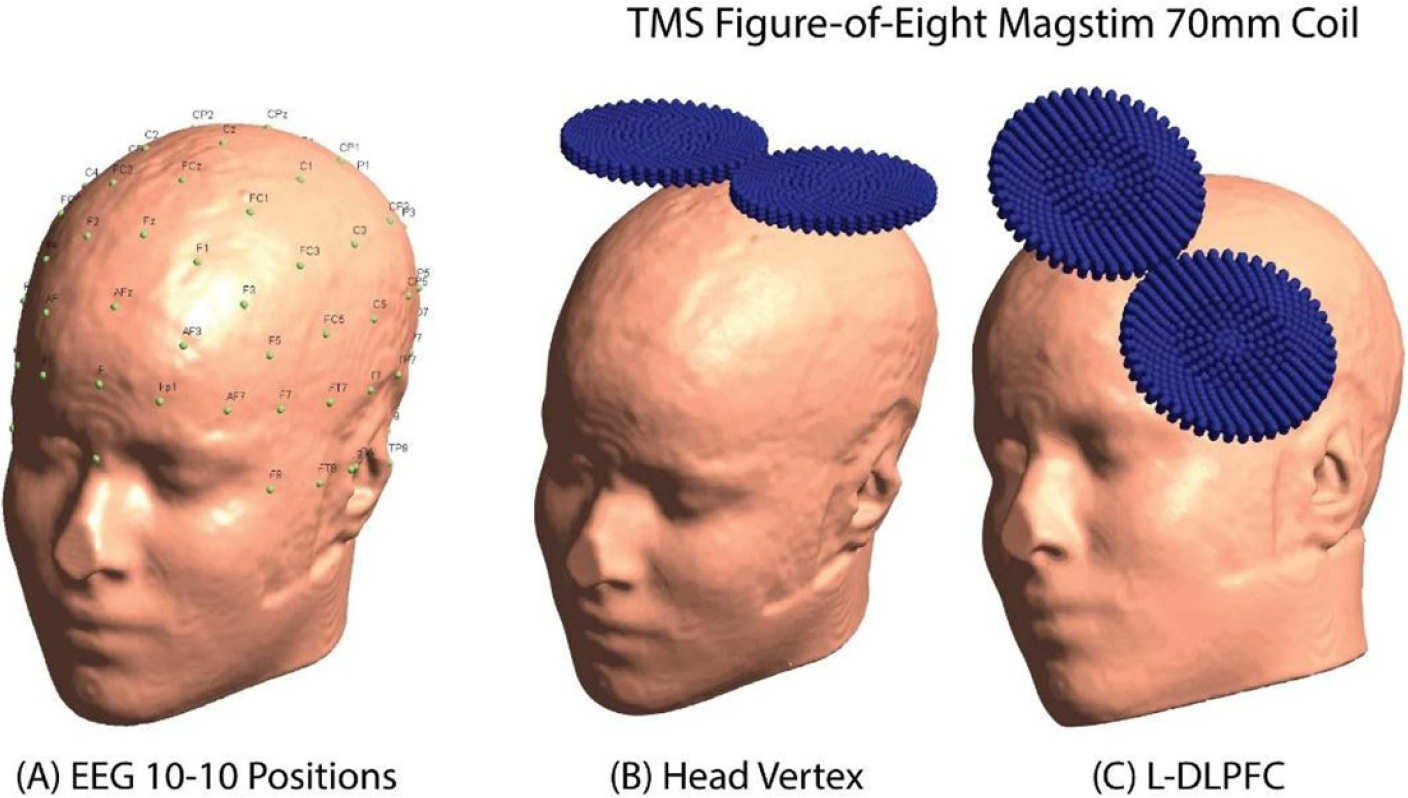
EEG electrodes positions on the scalp and TMS coil’s placements. (A) EEG 10–10 electrodes positions. (B) Head vertex coil placement at Cz electrode’s position. (C) L-DLPFC coil placement at F3 electrode’s position.

The placement locations of the TMS coil over the head followed electroencephalogram (EEG) 10–10 electrodes positions [41]. The EEG positions were pre-calculated using nonlinear transformation from the standard Montreal Neurological Institute (MNI) space to the individualized subject space which was latter projected on the subject’s scalp [33]. In this study, TMS stimulation was simulated on two brain regions: L-DLPFC and vertex, which is centered on the F3 and Cz EEG electrode positions, respectively [42, 43] (figure 2). The L-DLPFC was reported to be a major cortical region associated with cognitive [44] and emotional abnormalities [45, 46] and more dominantly used for depression therapy [47]. Vertex region on stimulation was utilized as control condition [28, 48, 49]. The coil placement orientation was standardized into the model anterior–posterior orientation and the coil’s major axis perpendicular to the model coronal plane. The coil center was placed 2 mm above the scalp to consider the hair thickness and scalp residue [23]. Besides, the coil placement was done on motor cortex (C3) to compare the vertex results for electric field peaks (Peak-EF) on all tissues.

### 2.4. Data post-processing and visualization methods

**Figure 3.**
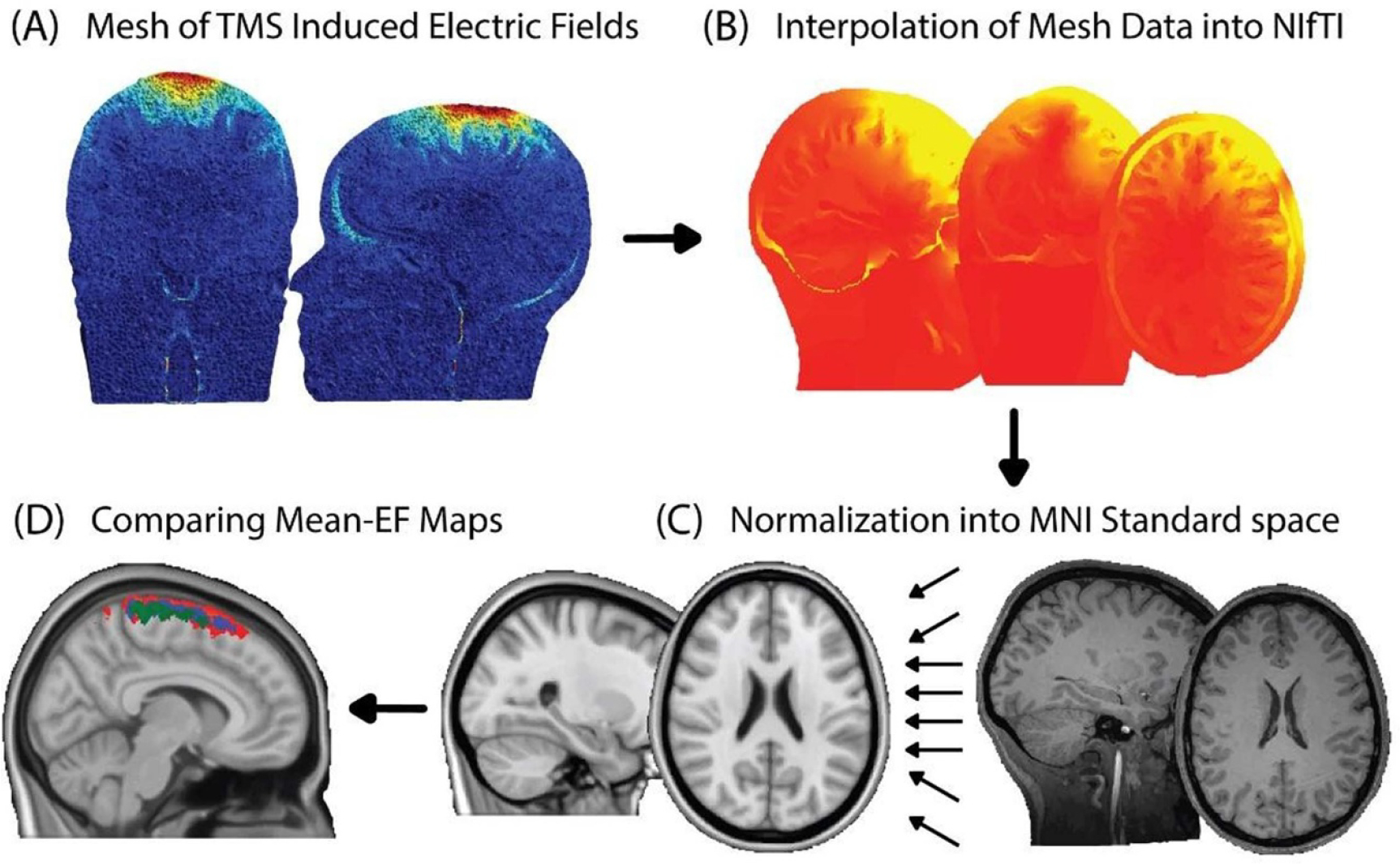
TMS-induced electric field distribution processing and visualization (A) collection of induced electric fields peak values. (B) Interpolation of the simulation volume mesh into NIfTI format. (C) Normalization the NIfTI format field into MNI space. (D) Calculating and comparing the MNI mean EF-map for each age group, children (red), adults (blue) and elderly (green).

The strength of the TMS induced electric field vectors was calculated and its peak strength (Peak-EF) identified in each tissue. The electric field magnitude was reportedly a stronger marker for neuronal activation than the normal component of the electric field [50]. To avoid computational outliers, the Peak-EF was considered as the 99.9th percentile of the TMS induced electric field distribution. For the TMS induced electric fields’ distribution analysis, the simulation results were translated from the mesh format into the Neuroimaging Informatics Technology Initiative (NIfTI) format by interpolating the electric fields values in the mesh tetrahedrons into an electric field map (EF-Map) [33] (figure 3). Thereafter, to allow group compression, the subjects EF-Maps were transformed to the standardized MNI space by determining the field deformation based on each individual T1-weighted scan. The MNI152 was used for all subjects’ normalization to avoid any data inconstancy or systematic differences resulted from the usage of different atlases. The mean EF-Maps in WM and GM were then calculated using SPM12, by averaging the EF-Maps of all the subjects within the age group and visualized using FSL software (figure 3).

### 2.5. Anatomical characteristics calculation method

To account for the age-related anatomical differences, the volumes of the white matter (WM_Vol_), grey matter (GM_Vol_), cerebrospinal fluid (CSF_Vol_) and the total intracranial volume (TIV) were calculated from their respective mesh tetrahedrons. The skull thickness and extra-axial CSF space thickness under the both TMS simulation placement were measured, by first viewing the subject’s T1-weighted image in Freeview software and then overlaying the bone, CSF and GM volume masks. Subsequently, the coil center coordinates were determined on the images to measure the perpendicular distances between the different masks in millimeter (mm) using the software’s Ruler tool. The masks’ coronal slice at the coil center was used to calculate the tissues thicknesses at the L-DLPFC. The skull thickness was measured between outer edge of the skull mask and CSF mask, while the extra-axial CSF space thickness was the distance between the outer edge of the CSF and GM mask. Similar steps were repeated at the vertex using a sagittal slice for the measurements.

### 2.6. Statistical analysis methods

Distribution normality was determined by Kolmogorov–Smirnov’s test and the equality of variances was tested using Levene’s test. ANOVA were used to explore the main effect of age on tissues characteristics (WM_Vol_, GM_Vol_, CSF_Vol_, TIV, extra-axial space (EAS) thickness and skull thickness) and the induced Peak-EF. ANOVA was followed by a series of *independent t-test* to investigate the differences between age groups pairwise, *Holm–Sidak post hoc* measures were adopted to correct for multiple comparisons. *Paired T-test* was used to explore the coil placement location effects Peak-EF within each tissue type. The standard statistical difference was considered significant at *P <* 0.05. *Pearson’s correlation test* was implemented to investigate the linear relationship between Peak-EF, age and other anatomical factors. *Python 3* libraries was used to perform the statistical analyses (Python Software Foundation, www.python.org/).

## 3. Results

### 3.1. Study sample demographic

The head model of the subjects was obtained from the link (attached in the supplementary file). T1-weighted anatomical scans for 60 subjects picked randomly form a pool of data. Subsequently, 12 subjects were excluded due to different segmentation and modeling errors associated with MRI’s facial features removal for privacy reasons by the original study. This method was employed by referring to a publication which reported on detailed modeling method for transcranial direct-current simulation induced E-Fields in children and adults [6]. The MRI scans quality reported by CAT12 showed mean resolution of 85% and weighted average image quality rating of 84.9%. The final successfully segmented head models consisted of 14 children (range = 4.0–12.0, median = 6.0 years), 19 adults (range = 22.0–37.0, median = 26.0 years) and 15 elderly (range = 75.0–84.0, median = 77.0 years). The age groups demographic shown in (table 2). The supplementary file describes the dataset in greater detail.

### 3.2. Anatomical tissues characteristics

**Figure 4.**
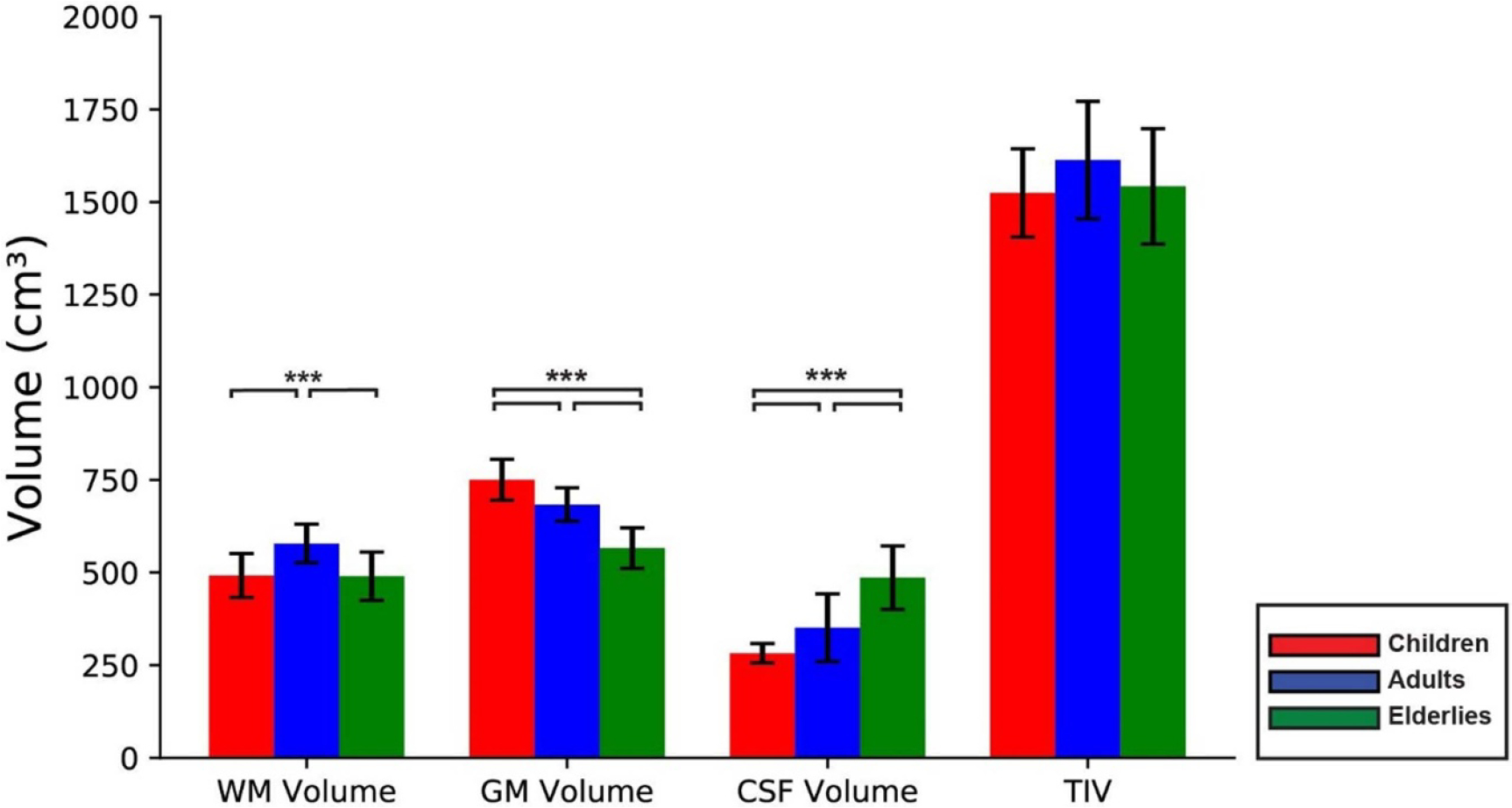
Brain tissues volume’s mean and standard deviation in children (red), adults (blue) and elderly (green) including the pairwise significant differences. ^∧^ * = *P* < 0.05, ** = *P* < 0.01 and *** = *P* < 0.001. WM (white matter); GM (grey matter); CSF (cerebrospinal fluid) and TIV (total intercortical volume).

**Figure 5.**
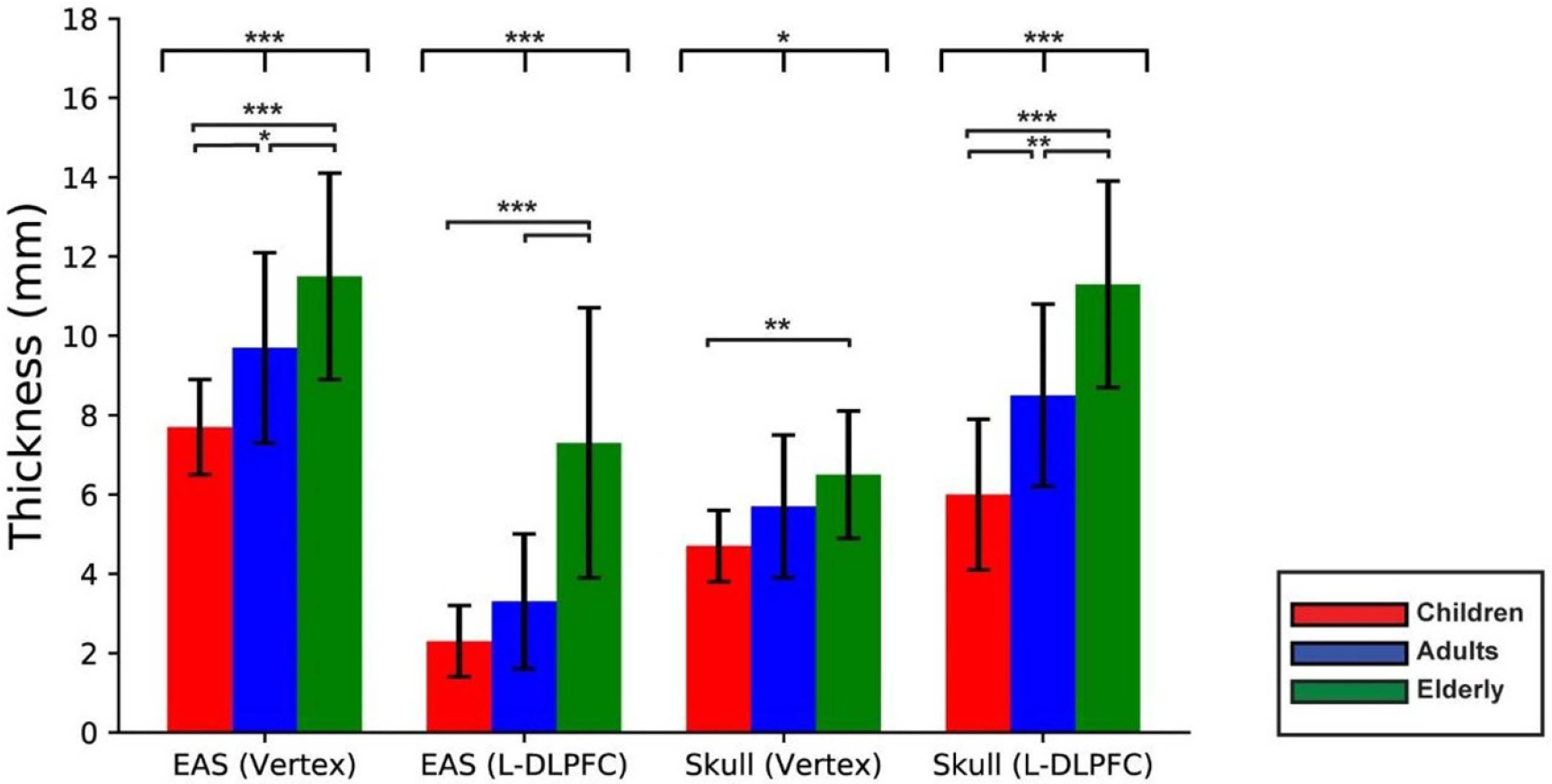
Extra-axial space (EAS) and skull thicknesses’ mean and standard deviation in children (red), adults (blue) and elderly (green) at two simulations regions (vertex and L-DLPFC) including the computed between groups (top) and pairwise (bottom) significant differences. ^∧^ * = *P* < 0.05, ** = *P* < 0.01 and *** = *P* < 0.001. EAS (extra-axial space); L-DLPFC (left dorsolateral prefrontal cortex).

Tissue characteristics of each age group are summarized in figures 4 and 5. The WM_Vol_ exhibited significant difference between the age groups (*P <* 0.001). The WM_Vol_ was significantly the highest in adults in comparison to children and elderly (both *P <* 0.001). There was no significant difference in the WM_Vol_ between children and elderly (*P* = 0.93). The WM_Vol_ and age did demonstrate a statistically significant linear correlation. The GM_Vol_ revealed significant difference between the age groups (*P <* 0.001). The GM_Vol_ was significantly the highest in children in comparison to adults and elderly (both *P <* 0.001) and significantly higher in adults in comparison to elderly (*P <* 0.001). The GM_Vol_ and age exhibited a significantly inverse linear correlation (*r* =−0.81, *P <* 0.001) (figure 4). The CSF_Vol_ showed a significant difference between the age groups (*P <* 0.001). The CSFVol was highest in elderly for age range of 75–84 years old in comparison to children with the age range between 4 and 12 years old and adults with age range between 22 and 37 years old (both *P <* 0.001) and significantly higher in adults in comparison to children (*P <* 0.001). The CSF_Vol_ and age revealed a significant direct correlation (*r* = 0.75, *P <* 0.001). Intercortical volume did not experience any significant variation between age groups (*P* = 0.191), with no significant correlation with age as well (figure 4).

The EAS under L-DLPFC was significantly different the between elderly in comparison to children and adults (both *P <* 0.001), but it had no significant variability between adults and children (*P* = 0.10). EAS thickness showed positive linear correlation with age under both simulation locations, vertex (*R* = 0.55, *P <* 0.001) and L-DLPFC (*r* = 0.71, *P <* 0.001). The EAS thickness was significantly larger at the vertex which serves as a control region to L-DLPFC in this study for all age groups (*P <* 0.01) (figure 5). Meanwhile, the EAS thickness demonstrated significant difference among the age groups at both the control region at vertex and L-DLPFC simulation positions (*P <* 0.001). Between age group pairs, independent *T* test revealed significant difference between the thickest EAS under the vertex of the elderly for age range of 75–84 years old in comparison to children with the age range between 4 and 12 years old (*P <* 0.001) and adults with age range between 22 and 37 years old (*P* = 0.04), and in the EAS of adults compared to children (*P* = 0.016).

The skull thickness showed a significant variation between the age groups, specifically under the L-DLPFC (*P <* 0.001) and vertex (*P* = 0.013) between the age group pairs, the skull thickness under the vertex was significantly higher in elderly with age range 75–84 years old in comparison to children only (*P* = 0.004), in addition to an insignificant difference between adults with age range between 22 and 37 years old and children with age range of 4–12 years old (*P* = 0.13). At the L-DLPFC, the highest skull thickness in elderly was obtained a significant different in comparison to adults (*P* = 0.003) and children (*P <* 0.001), in addition to significant difference between children and elderly toward the skull thickness in the latter (*P* = 0.003). Figure 5 shows the elderly has the thickest skull in compared to adults and children for both L-DLPFC and vertex.

### 3.3. Strength of TMS induced electric fields

**Figure 6.**
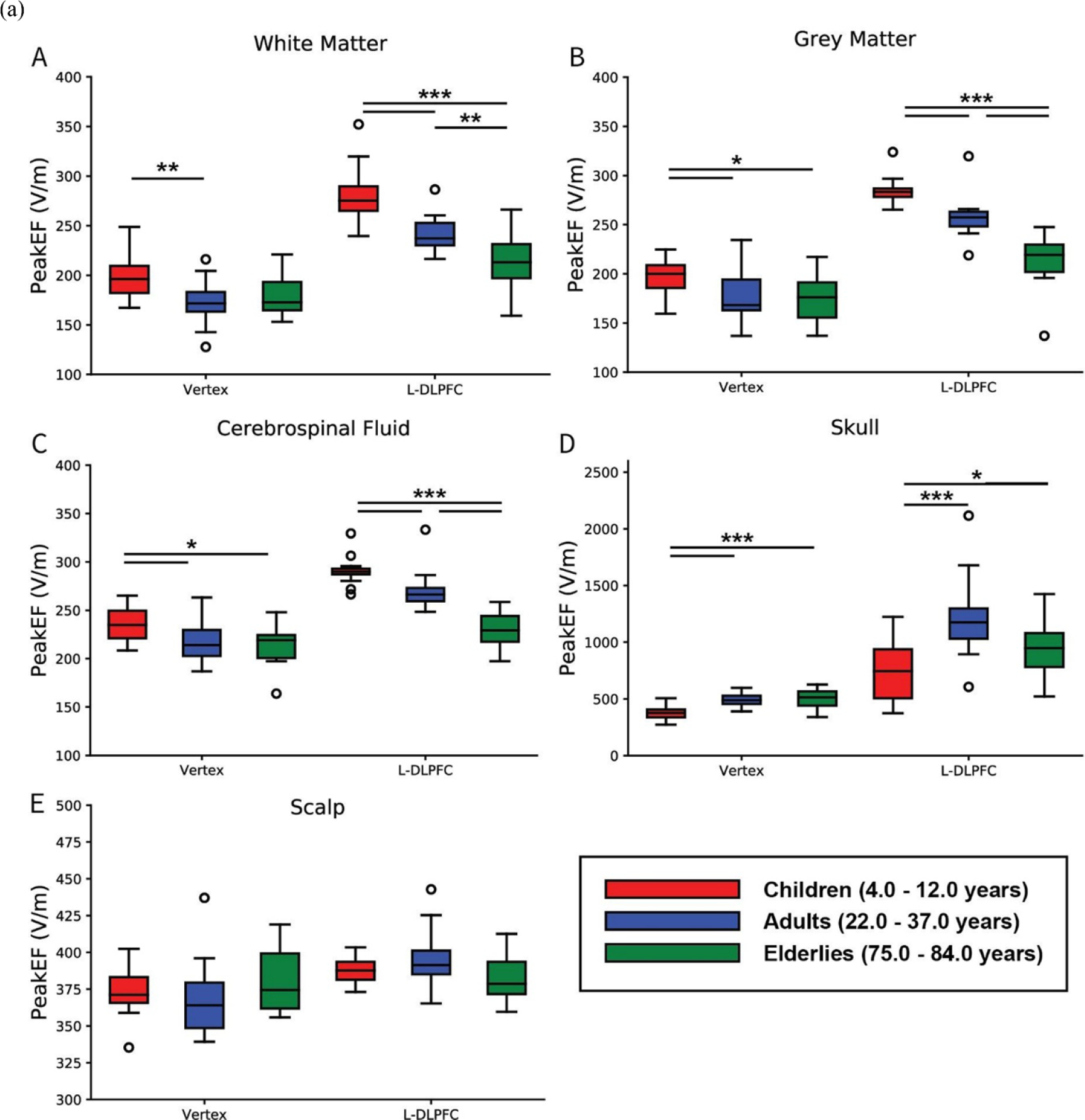

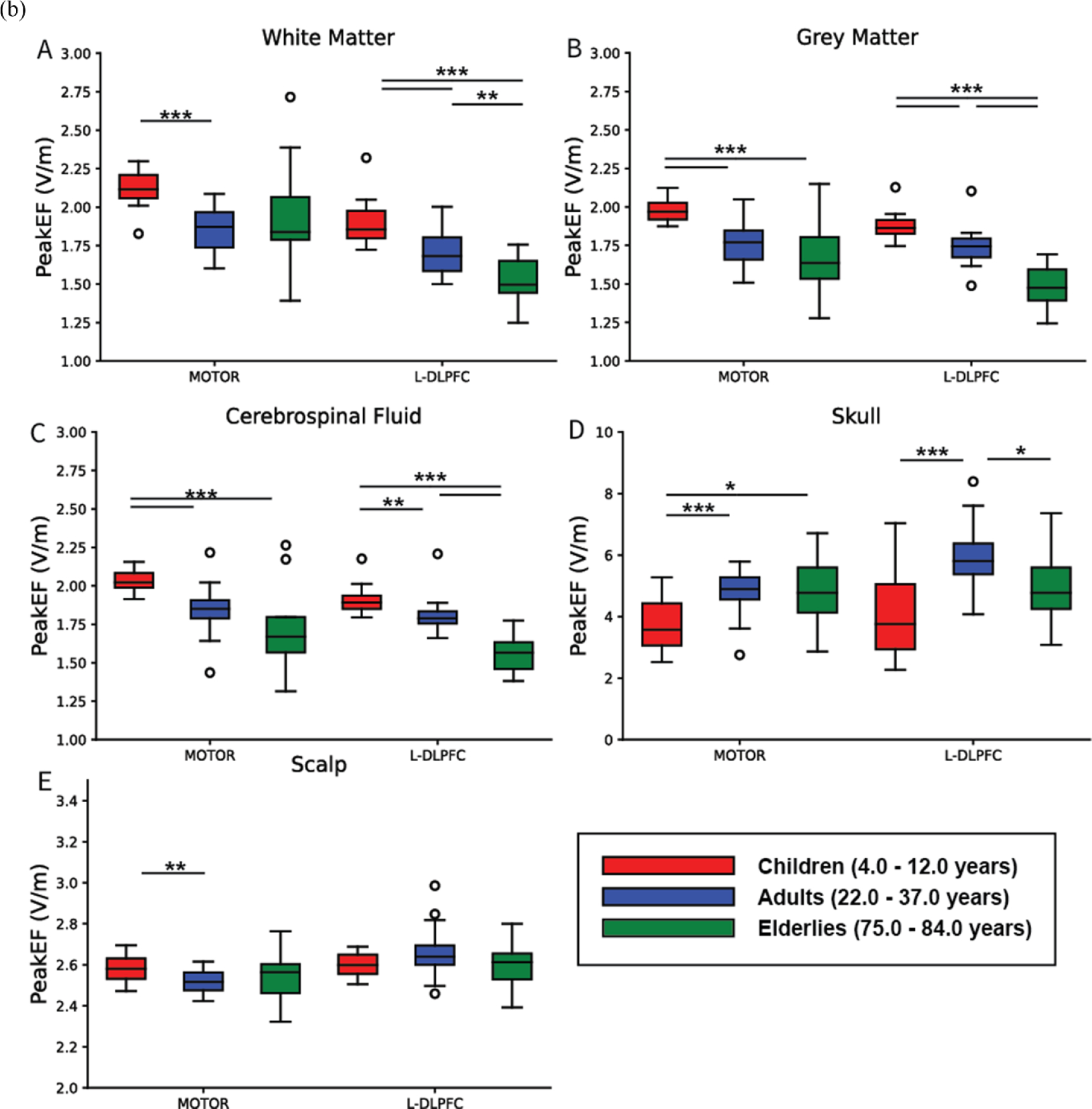
(a) The peak electric field induced by TMS in children (red), adults (blue) and elderly (green) different tissues at two simulations regions (vertex and L-DLPFC) including the pairwise significant differences. ^∧^ * = *P* < 0.05, ** = *P* < 0.01 and *** = *P* < 0.001. EAS (Extra-Axial Space); L-DLPFC (left dorsolateral prefrontal cortex). (b) The peak electric field induced by TMS in children (red), adults (blue) and elderly (green) different tissues at two simulations regions (motor cortex (c3) and L-DLPFC) including the pairwise significant differences. ∧ * = *P* < 0.05, ** = *P* < 0.01 and *** = *P* < 0.001. EAS (Extra-Axial Space); L-DLPFC (left dorsolateral prefrontal cortex). *Y* scale: *×*10^2^.

The TMS induced Peak-EF in the five tissue types at both simulation locations were summarized in (figure 6). TMS at the vertex induced a significantly different Peak-EF in all tissues between the age groups (all *P <* 0.018), except for the scalp (*P* = 0.284). The children’s WM reveals a significantly higher Peak-EF than adults (*P* = 0.004), but not elderly (*P* = 0.052). The children’s GM and CSF revealed a significantly higher Peak-EF (both *P <* 0.04) and lower Peak-EF in children’s skull (*P <* 0.001) in comparison to adults and elderly. In contrast, the adults and elderly groups did not record any significant difference in all tissues (figure 6). The Peak-EF had an inverse linear correlation with age in the GM and CSF (*P <* 0.03) and a positive correlation with age in the skull (*P* = 0.001), without any significant correlations between the Peak-EF and age in the WM and scalp.

The Peak-EF at the L-DLPFC showed similar patterns with a significantly different Peak-EF among the age groups in all tissues (*P <* 0.001) except for skin (*P* = 0.104). The Peak-EF was the highest in children with age range of 4–12 years old WM, GM and CSF (all *P <* 0.001) and significantly the lowest in the children skull (*P* < 0.03) in comparison to adults and elderly. On the other hand, the Peak-EF was significantly higher in the adults with age range between 22 and 37 years old for their GM, WM and CSF (all *P* < 0.003) and significantly lower in the adult’s skull (*P* = 0.031) in comparison to elderly group (figure 6(a)). The results suggested a strong negative correlation between the Peak-EF in WM, GM and CSF with increasing age (*P* < 0.001), with insignificant correlation with age in the skull and scalp. On the other hand, figure 6(b) shows the Peak-EF at the motor region (C3) as another additional reference or standard control region to the vertex displaying the same pattern for all five-tissue type except for scalp. In the motor cortex there were significant differences between the children and adult groups, but this was not the case in the vertex and L-DLPFC.

The Peak-EF correlation coefficients with age-related anatomical factors (table 3), for instance, the skull and extra-axial CSF space thickness, the CSF volume and intracranial volume were measured. At the L-DLPFC simulation location, The Peak-EF in the WM, GM and CSF tissues experienced an inverse linear correlation with the skull thickness (all *P* <0.001). Nevertheless, at the vertex location, the Peak-EF in the CSF tissues showed a significant inverse linear correlation with skull thickness (*r* = −0.361, *P* = 0.011) as well. Moreover, the inverse linear correlations between the Peak-EF and EAS thickness in the WM, GM and CSF tissues indicated drop in the induced Peak-EF with the increase in the EAS thickness in both stimulation locations. Conversely, the Peak-EF and TIV did not show any significant correlation. Additionally, in all tissues, the Peak-EF in the L-DLPFC showed significantly stronger TMS induced fields in comparison to the vertex, except in the scalp tissue of the elderly group with age range of 75–84 years old.

## 4. Discussion

This study modeled the TMS induced electric fields among three age groups to examine variability in electric field distributions. Models of children had higher field strengths and wider distributions of TMS induced electric fields compared to the adult and elderly groups. The TMS induced electric field in the elderly demonstrated the weakest field strength and narrowest spread among all groups. The results at the L-DLPFC would suggest that the thin skull in the children group may contribute to the variability in electric fields in this age group compared to others [51]. As a result, TMS dosing may have a greater stimulation strength in children than anticipated. In contrast, the thicker skull in the adult’s group and the thickest skull in elderly group gave converse effects which have led to a weaker TMS induced electric fields in the inner tissues (WM, GM and CSF) [51, 52]. These findings were further supported by the significant inverse linear correlation between the skull thickness and the Peak-EF generated in WM, GM and CSF [52]. The vertex region had a similar trend. Modeling on the conductivity of the compact and trabecular bones of skull structure on its conductivity with EEG suggested that the local variations over the skull surface are for both isotropic and anisotropic skull conductivity has little influence [53]. Another modeling study compared on a realistic head and spherical model suggests an interesting point that it is essential to measure the skull conductivity of the individual patients to achieve accurate EEG source analysis [54]. Meanwhile, age-related bone loss in the elderly population leads to morphological changes in the diploe of the human skull [55, 56] might receive different impacts from TMS electric field.

The present findings suggest that Peak-EF was not only influenced by the skull thickness but also by other age-related anatomical factors such as thickness of EAS and the variation of WM and GM geometries beneath the simulation site [57, 58]. In this case, it would be desirable to compare between EAS thickness and the induced electric field at the two simulation regions as well. The EAS appeared to be the thinnest in the children and thickest in the elderly, alongside being thicker at the control region of vertex in comparison to the L-DLPFC region. The thinner EAS reportedly contributed to amplification of the TMS fields that reaches the brain’s WM and GM, this was due to the formation of strong local Peak-EF at the tissues boundaries resulted from the electrical disassociation between high conductivity of the CSFs and the lower conductivity of the GM tissues [59]. On the other hand, the thicker CSF has dropped the tendency of local maximum peaks formation, hence the TMS fields amplifying effects too. This fore the reason that the electric field would spread widely in CSFs reducing the effects of conductivities variation at the tissues CSF/GM boundary and therefore the amount of induced electric field in the brain’s WM and GM [57]. These findings were further supported by the found inversely linear correlation between the EAS thickness and the Peak-EF induced in WM, GM and CSF at both TMS simulation locations. Conjointly, these relationships between skull and EAS thicknesses and the TMS induced electric field could establish a similarity with previous studies findings that related the increase in scalp-to-cortex distance (skull thickness + extra-axial thickness) to the drop of TMS fields that reaches the WM and GM consequently, reduce the TMS induced electric field in these tissues [51]. The scalp-to-cortex distance effect is further support in this study showing that smaller scalp-to-cortex distance leads to higher TMS induced electric fields in the children, and that larger scalp-to-cortex distance results in higher field attenuation and lower TMS induced electric fields in the elderly. It should be noted that, the children younger than the modeled sample (age *<* 4) were reported having thinner skull [53]. Hence, this makes the scalp-to-cortex distance even shorter. Furthermore, patients who undergo skull craniotomy might experience significantly higher TMS induced electric fields in compared to the patients with ordinary skull [54, 55]. Here, it could be argued that the scalp-to-cortex distance influences on the TMS induced fields could be eliminated by assessing the MEP threshold over the motor cortex. A prior study shows the quantified motor threshold depends on coil-to-cortex distance (CCD), and suggests that the computed electric field could be used as a measure to decrease the within- subject effect of CCD [56].

Differences between the induced fields could be seen in all age groups with respect to comparison stimulation site. The simulation parameters at both regions were identical and the only variable was the TMS coil’s position. Generally, the induced fields at L-DLPFC have shown significantly higher strength in compared to the vertex region. These findings suggested the variabilities in TMS induced electric field at different simulation regions have emerged from the anatomy geometrical differences underneath the TMS coil [23, 60, 61].

In addition to the age-related effects on the induced fields’ strengths, this study indicated similar age-related influences on the induced fields’ distribution and depth. The TMS induced fields in the children group showed the most expansive distribution in the brain tissues in comparison to adults and elderly groups. In contrary, the elderly group showed the least expansive distribution. However, a review related that elderly patient are often excluded from TMS protocols due to the absence of evidence in this age group. However, findings are mixed with respect to the utility of the TMS for elderly patients with depression [61]. A recent study on localizing analysis of normalized distance from scalp to cortex on age and dementia-specific changes suggested that both groups showed different impacts on scalp to cortex distance of L-DLPFC and left primary motor cortex in conducting neuromodulation for different individual with old age and dementia [62]. Quantitative synthesize review with TMS examination on cortical excitability and plasticity in the human brain suggested that a reduction in age-related differences in cortical excitability and sensorimotor integration within the human motor cortex [31]. EF-Peak has done on motor cortex at C3 to compare with the sham region (vertex) which displays the similar pattern across age group for all types of tissues modeled in this work except scalp which the pairwise significant between children and adults are significant. Prior report shows that skull-to-cortex distance and the anterior component of the principal diffusion direction of the corticospinal tract are predictive as TMS motor threshold in a linear regression model [63]. On the other hand, one report indicates the CCD directly effects the magnitude of cortical simulation in TMS. It was reporting on the success of mapping TMS on motor cortex in awake children who were younger than three years of age [64]. CCD directly influences the magnitude of cortical stimulation in TMS and provided a simple and effective method for scaling stimulator output to a distance adjusted motor threshold [65].

This study has limitations that should be considered in the context of interpreting the data. Simulations and modeling work require assumptions. For instance, the conductivities of the tissues were not measured in this study, rather practical approximation were taken from previously studies [38], additionally, negligent of the eddy-current effects by assuming the coil as set of isolated dipole elements might results in small numerical error [39]. In regards of the EEG electrode positioning in SimNIBS the author has reported 2–8 mm error for the true electrode position. However, this error was below the average at the vertex (C_Z_) and L-DLPFC (F3). This error could be considered acceptable as in this study the anatomical distance between the vertex and the L-DLPFC is greatly larger in compared to the positioning error [66]. On the tissue segments quality in SimNIBS, the author reported that the usage of the T2 MRI sequence besides the T1 would improve the skull geometry, but due the absence of the T2 MRI in this study dataset, manual segments verification was used to ensure the tissue segments accuracy [66]. Furthermore, a wide spared of age-span to spot age-related differential effects on the TMS induced electric fields, it was not powerful enough to reveal any effects of gender on the TMS induced fields or to figure higher-order relationships between the variables at a cubic or quadratic level, in addition, *F* test analysis is worthy to add in for obtaining a more reliable modeling. Another limitation of this work is on the finally, there are factors not modeled in this study that can affect physiological response to the induced electric fields. For example, it has been reported that motor threshold—the minimum TMS intensity required to elicit a MEP of a certain magnitude—is a decreasing function of age, up to an adult age of 35 [64]. This observation seemingly contradicts our finding of higher induced electric field in the child brain compared to the adult brain. The negative relationship between age and motor threshold may reflect maturation process of axonal myelination or of GABAergic interneurons [64]. The modeling of these physiological processes is well beyond the scope of this work primarily focusing on electric field modeling. However, pediatric researchers should caution that the higher electric field in children, combined with higher physiological thresholds compared to adult, could be of a safety concern in TMS studies involving children. Although, report shows that no empirical evidence suggested single or paired pulse TMS brings more than minimal risk in children [66], however, additional study is still desired to unveil the significance of found statistical differences in the clinical settings.

## 5. Conclusion

In a conclusion, the age-related anatomical changes and the anatomical geometry beneath the simulation’s coil carried significant impact on the TMS induced electric fields in different age groups. This study calls for safety precautions for TMS applications and protocols for children in specification as the EF-Map in the children showed the widest cortical surface spread of TMS induced electric fields among another group. Contrary, the smallest cortical surface spread in elderly might suggest the increase in TMS induced electric field for more effective therapeutic effect for this age group. In the future, further investigations might be needed using the TMS fields’ modeling techniques to design personalized diagnostic or therapeutic interventions for TMS applications in different age groups.

## Supplementary Material

Supplementary Material

## Figures and Tables

**Table 1.** Tissues conductivity values [48, 49].

Tissue Type	Conductivity Value Siemens/meter (S m^−1^)

WM	0.126
GM	0.275
CSF	1.654
Skull	0.010
Scalp	0.465

**Table 2.** Study sample’s demographic data based on three age groups (children, adults and elderly).

Population	Age range	Male	Female

Children	4–12	7	7
Adults	22–37	9	10
Elderly	75–84	6	9

**Table 3.** Correlation of peak-EF with different anatomical factors.

Tissue type	Coil Location	Anatomical factor					
		Skull thickness		Extra-Axial space thickness		TIV	

		*r*	*p*-value	*r*	*p*-value	*r*	*p*-value

WM	Vertex	−0.1836	0.21	−0.210	0.016*	−0.056	0.71
	L-DLPFC	−0.6742	<0.001***	−0.385	<0.001***	0.095	0.52
GM	Vertex	−0.1845	0.21	−0.410	<0.001***	−0.132	0.37
	L-DLPFC	−0.6743	<0.001***	−0.544	<0.001***	0.073	0.62
CSF	Vertex	−0.361	0.012*	−0.289	0.002**	0.011	0.94
	L-DLPFC	−0.761	<0.001***	−0.518	<0.001***	0.096	0.52
Skull	Vertex	−0.113	0.44				
	L-DLPFC	−0.200	0.17				
Scalp	Vertex						
	L-DLPFC						

^ * = *p* < 0.05, ** = *p* < 0.01 and *** = *p* < 0.001.

*r* (Pearson’s correlation coefficient); WM (white matter); GM (grey matter); CSF (cerebrospinal fluid); L-DLPFC (left dorsolateral prefrontal cortex).

## Data Availability

All data that support the findings of this study are included within the article.
