# Supplementary material for: Modelling the differential effects of age on transcranial magnetic stimulation induced electric fields
Source: J Neural Eng. Author manuscript; Available in PMC 2023 Jun 19. (PMC10278869; doi:10.1088/1741-2552/ac9a76)
Supplement: Supplementary Material [file NIHMS1905725-supplement-Supplementary_Material.docx]

Supplementary Material

# Study dataset

| Dataset no. | Title | Number of Subjects | Online Link |
| --- | --- | --- | --- |
| 1 | Behavioral interventions for reducing head motion during MRI scans in children | 24 | <https://openneuro.org/datasets/ds000256/versions/00002> |
|  |  |  |  |
| 2 | Balloon Analog Risk Task | 16 | <https://openneuro.org/datasets/ds000001/versions/1.0.0> |
|  |  |  |  |
| 3 | MRI data of 3-12 year old children and adults during viewing of a short animated film | 155 | <https://openneuro.org/datasets/ds000228/versions/1.1.0> |
|  |  |  |  |
| 4 | Neural Processing of Emotional Musical and Nonmusical Stimuli in Depression | 39 | <https://openneuro.org/datasets/ds000171/versions/00001> |
|  |  |  |  |
| 5 | Preschool Anxiety Disorders | 45 | <https://openneuro.org/datasets/ds000144/versions/00002> |
|  |  |  |  |
| 6 | Resting State Perfusion in Healthy Aging | 63 | <https://openneuro.org/datasets/ds000240/versions/00002> |
|  |  |  |  |
| 7 | Olfactory dysfunction and functional connectivity changes in cognitively normal Parkinson’s disease | 45 | <https://openneuro.org/datasets/ds000245/versions/00001> |
| 8 | Whole-brain background-suppressed pCASL MRI with 1D-accelerated 3D RARE Stack-Of-Spirals Readout- Dataset 3 | 16 | <https://openneuro.org/datasets/ds000236/versions/2.0.1> |

**Supplementary Table 1.** The Downloaded Datasets to Create the Data Pool Used for the Study Sample Selection.

# Study Sample

| Subject ID in the current study | Origin Dataset no. | Subject ID in the origin dataset | Age  (years) | Gender | Group |
| --- | --- | --- | --- | --- | --- |
| SUB_1 | 3 | sub-pixar023 | 4.0 | F | Children |
| SUB_2 | 3 | sub-pixar031 | 4.1 | F | Children |
| SUB_3 | 3 | sub-pixar005 | 4.8 | F | Children |
| SUB_4 | 3 | sub-pixar002 | 4.9 | F | Children |
| SUB_5 | 3 | sub-pixar036 | 5.4 | F | Children |
| SUB_6 | 3 | sub-pixar059 | 5.5 | F | Children |
| SUB_7 | 3 | sub-pixar061 | 5.6 | F | Children |
| SUB_8 | 5 | sub-11 | 7.0 | F | Children |
| SUB_9 | 5 | sub-17 | 7.0 | F | Children |
| SUB_10 | 3 | sub-pixar113 | 10.3 | F | Children |
| SUB_11 | 3 | sub-pixar051 | 5.4 | M | Children |
| SUB_12 | 3 | sub-pixar041 | 5.5 | M | Children |
| SUB_13 | 5 | sub-28 | 6.0 | M | Children |
| SUB_14 | 3 | sub-pixar079 | 7.1 | M | Children |
| SUB_15 | 3 | sub-pixar080 | 7.9 | M | Children |
| SUB_16 | 3 | sub-pixar069 | 8.0 | M | Children |
| SUB_17 | 5 | sub-14 | 8.0 | M | Children |
| SUB_18 | 3 | sub-pixar091 | 9.7 | M | Children |
| SUB_19 | 3 | sub-pixar098 | 10.2 | M | Children |
| SUB_20 | 3 | sub-pixar117 | 12.3 | M | Children |
| SUB_41 | 3 | sub-pixar132 | 22.0 | F | Adults |
| SUB_42 | 3 | sub-control13 | 22.0 | F | Adults |
| SUB_43 | 2 | sub-11 | 24.0 | F | Adults |
| SUB_44 | 3 | sub-pixar149 | 24.0 | F | Adults |
| SUB_45 | 6 | sub-02 | 24.0 | F | Adults |
| SUB_46 | 3 | sub-mdd07 | 26.0 | F | Adults |
| SUB_47 | 6 | sub-03 | 26.0 | F | Adults |
| SUB_48 | 3 | sub-pixar123 | 27.1 | F | Adults |
| SUB_49 | 6 | sub-20 | 29.0 | F | Adults |
| SUB_50 | 3 | sub-control16 | 37.0 | F | Adults |
| SUB_51 | 6 | sub-15 | 22.0 | M | Adults |
| SUB_52 | 2 | sub-02 | 24.0 | M | Adults |
| SUB_53 | 6 | sub-05 | 26.0 | M | Adults |
| Subject ID in the current study | Origin Dataset no. | Subject ID in the origin dataset | Age  (years) | Gender | Group |
| SUB_54 | 3 | sub-pixar128 | 27.0 | M | Adults |
| SUB_55 | 4 | sub-control05 | 27.0 | M | Adults |
| SUB_56 | 3 | sub-pixar154 | 29.0 | M | Adults |
| SUB_57 | 4 | sub-mdd09 | 30.0 | M | Adults |
| SUB_58 | 3 | sub-pixar148 | 31.0 | M | Adults |
| SUB_59 | 4 | sub-control06 | 31.0 | M | Adults |
| SUB_60 | 6 | sub-27 | 38.0 | M | Adults |
| SUB_103 | 8 | sub-17 | 75.0 | F | Elderly |
| SUB_104 | 7 | sub-ODN10 | 75.0 | F | Elderly |
| SUB_105 | 7 | sub-ODP13 | 75.0 | F | Elderly |
| SUB_106 | 8 | sub-11 | 77.0 | F | Elderly |
| SUB_107 | 8 | sub-12 | 77.0 | F | Elderly |
| SUB_108 | 7 | sub-ODP15 | 78.0 | F | Elderly |
| SUB_109 | 6 | sub-34 | 78.0 | F | Elderly |
| SUB_110 | 6 | sub-60 | 78.0 | F | Elderly |
| SUB_111 | 6 | sub-52 | 82.0 | F | Elderly |
| SUB_112 | 6 | sub-15 | 84.0 | F | Elderly |
| SUB_101 | 8 | sub-16 | 75.0 | M | Elderly |
| SUB_102 | 7 | sub-ODN12 | 75.0 | M | Elderly |
| SUB_113 | 8 | sub-03 | 75.0 | M | Elderly |
| SUB_114 | 8 | sub-07 | 75.0 | M | Elderly |
| SUB_115 | 6 | sub-55 | 75.0 | M | Elderly |
| SUB_116 | 6 | sub-54 | 76.0 | M | Elderly |
| SUB_117 | 6 | sub-43 | 77.0 | M | Elderly |
| SUB_118 | 6 | sub-63 | 77.0 | M | Elderly |
| SUB_119 | 6 | sub-56 | 78.0 | M | Elderly |
| SUB_120 | 6 | sub-46 | 81.0 | M | Elderly |
|  | Segmentation error | | | | |
|  | Bad segmentation quality | | | | |

**Supplementary Table 2.** The Selected Study Sample Given the Subject ID in the Current Study and in the Origin Dataset with the Age, Gender and Group of each Subject, Highlighted the Excluded Subjects with the Exclusion Reason.

# Statistical Significance Values

| Category | Participant Groups | | |
| --- | --- | --- | --- |
| Mean (SD) [Range or %] | Children | Adults | Elderlies |
| Age(years) | 6.9 (2.4)  [4.0 - 12.0] | 26.6 (3.9 )  [22.0 - 37.0] | 77.7 (2.8)  [75.0 - 84.0] |
| Sex % |  |  |  |
| Male | N = 7 [50%] | N = 9 [47.4%] | N = 6 [40%] |
| Female | N = 7 [50%] | N = 10 [52.6%] | N = 9 [60%] |
| Total | N = 14 | N = 19 | N = 15 |
| WM VOLUME (cm³) | 491.9 (59.3)  [434.2 - 616.9] | 578.2 (52.1)  [475.7 - 645.2] | 490.1 (65.0)  [363.6 - 657.9] |
| GM VOLUME (cm³) | 750.5 (55.0)  [656.5 - 866.3] | 683.5 (44.9)  [585.2 - 743.8] | 565.8 (54.6)  [496.4 - 723.7] |
| CSF VOLUME (cm³) | 282 (26.3)  [245.4 - 348.3] | 351.2 (91.1)  [269.9 - 640.2] | 486.3 (85.4)  [345.6 - 619.6] |
| TIV (cm³) | 1524.3 (119.4) [1379.2 - 1831.5] | 1612.9 (158.6) [1375.8 - 1980.1] | 1542.2 (155.6) [1337.2 - 1978.9] |
| Extra-Axial Space Thickness (mm) |  |  |  |
| Vertex | 2.2 (0.8) [1.18 - 9.8] | 3.1 (1.6) [1.2 - 15.0] | 7.0 (3.2) [2.5 - 14.0] |
| L-DLPFC | 2.3 (0.9) [1.2 - 4.3] | 3.3 (1.7) [1.3 - 6.2] | 7.3 (3.4) [2.7 - 12.6] |
| Paired T-test  (p-value) | 0.03* | < 0.001*** | 0.002** |
| Skull Thickness (mm) |  |  |  |
| Vertex | 5.8 (1.9) [3.0 – 4.2] | 8.3 (2.2) [5.3 – 5.8] | 11.0(2.5) [7.0- 6.7] |
| L-DLPFC | 6 (1.9) [3.1 - 11.1] | 8.5 (2.3) [5.6 - 13.3] | 11.3 (2.6) [7.3 - 16.2] |
| Paired T-test  (p-value) | < 0.001*** | < 0.001*** | < 0.001*** |

*^ * = ρ < 0.05, ** = ρ < 0.01 and *** = ρ < 0.001. . SD (Standard Deviation); Peak-EF (TMS Induced Electric Field Peak); WM (White Matter); GM (Grey Matter); CSF (cerebrospinal fluid); TIV (Total Intercortical volume); L-DLPFC (left dorsolateral prefrontal cortex).*

**Supplementary Table 3.** Subjects’ Demographic and Tissues Characteristics.

| Category | Between Groups | Pairwise | | |
| --- | --- | --- | --- | --- |
|  | ANOVA  p-value | Independent T-Test (p-value) | | |
|  |  | Children vs Adults | Children vs. Elderly | Adults vs. Elderly |
| Age | < 0.001*** | < 0.001*** | < 0.001*** | < 0.001*** |
| WM VOLUME | < 0.001*** | < 0.001*** | 0.937 | < 0.001*** |
| GM VOLUME | < 0.001*** | < 0.001*** | < 0.001*** | < 0.001*** |
| CSF VOLUME | < 0.001*** | < 0.001*** | < 0.001*** | < 0.001*** |
| TIV | 0.191 | 0.246 | 0.733 | 0.364 |
|  |  |  |  |  |
| Extra-Axial Space Thickness |  |  |  |  |
| MOTOR | < 0.001*** | 0.127 | < 0.001*** | < 0.001*** |
| L-DLPFC | < 0.001*** | 0.103 | < 0.001*** | < 0.001*** |
|  |  |  |  |  |
| Skull Thickness |  |  |  |  |
| MOTOR | < 0.001*** | 0.002** | 0.004** | 0.002** |
| L-DLPFC | < 0.001*** | 0.003** | < 0.001*** | 0.003** |

*^ * = ρ < 0.05, ** = ρ < 0.01 and *** = ρ < 0.001. WM (White Matter); GM (Grey Matter); CSF (cerebrospinal fluid); TIV (Total Intercortical volume); L-DLPFC (left dorsolateral prefrontal cortex).*

**Supplementary Table 4.** Statistical Analysis of Age Groups Demographic and Tissue Characteristics.

| TISSUE TYPE | COIL LOCATION | Between Groups | Pairwise | | |
| --- | --- | --- | --- | --- | --- |
|  |  | p -value | p -value | | |
|  |  |  | Children vs. Adults | Children vs. Elderlies | Adults vs. Elderlies |
| WM | MOTOR | 0.003** | < 0.001*** | 0.057 | 0.26 |
|  | L-DLPFC | < 0.001*** | < 0.001*** | < 0.001*** | 0.0014** |
| GM | MOTOR | 0.018* | < 0.001*** | < 0.001*** | 0.31 |
|  | L-DLPFC | < 0.001*** | 0.0018** | < 0.001*** | < 0.001*** |
| CSF | MOTOR | 0.013* | < 0.001*** | < 0.001*** | 0.63 |
|  | L-DLPFC | < 0.001*** | 0.008** | < 0.001*** | < 0.001*** |
| Skull | MOTOR | 0.003** | < 0.001*** | 0.011* | 0.99 |
|  | L-DLPFC | < 0.001*** | < 0.001*** | 0.107 | 0.018* |
| Scalp | MOTOR | 0.134 | 0.008** | 0.35 | 0.40 |
|  | L-DLPFC | 0.139 | 0.12 | 0.74 | 0.10 |

*^ * = ρ < 0.05, ** = ρ < 0.01 and *** = ρ < 0.001. WM (White Matter); GM (Grey Matter); CSF (cerebrospinal fluid); L-DLPFC (left dorsolateral prefrontal cortex).*

**Supplementary Table 5.** Statistical Analysis of Peak Electric Fields Induced by TMS in Different Age Groups.

| Tissue type | Coil Location | Age | |
| --- | --- | --- | --- |
|  |  |  |  |
|  |  | R | p-value |
| WM | MOTOR | -0.202 | 0.16 |
|  | L-DLPFC | -0.688 | < 0.001*** |
| GM | MOTOR | -0.513 | < 0.001*** |
|  | L-DLPFC | -0.809 | < 0.001*** |
| CSF | MOTOR | -0.555 | < 0.001*** |
|  | L-DLPFC | -0.800 | < 0.001*** |
| Skull | MOTOR | 0.326 | 0.02* |
|  | L-DLPFC | 0.068 | 0.64 |
| Scalp | MOTOR | -0.087 | 0.55 |
|  | L-DLPFC | -0.129 | 0.38 |

*^ * = ρ < 0.05, ** = ρ < 0.01 and *** = ρ < 0.001. WM (White Matter); GM (Grey Matter); CSF (cerebrospinal fluid); L-DLPFC (left dorsolateral prefrontal cortex).*

**Supplementary Table 6.** Correlation of Peak-EF with Age.

| Tissue type | Coil Location | Anatomical factor | | | | | |
| --- | --- | --- | --- | --- | --- | --- | --- |
|  |  | Skull  thickness | | Extra-Axial space thickness | | TIV | |
|  |  | R | p-value | R | p-value | R | p-value |
| WM | MOTOR | -0.467 | < 0.001*** | -0.129 | 0.38 | 0.008 | 0.95 |
|  | L-DLPFC | -0.648 | < 0.001*** | -0.570 | < 0.001*** | 0.173 | 0.23 |
| GM | MOTOR | -0.605 | < 0.001*** | -0.339 | 0.018* | 0.037 | 0.79 |
|  | L-DLPFC | -0.678 | < 0.001*** | -0.593 | < 0.001*** | 0.073 | 0.96 |
| CSF | MOTOR | -0.674 | < 0.001*** | -0.382 | 0.007** | 0.075 | 0.61 |
|  | L-DLPFC | -0.758 | < 0.001*** | -0.570 | < 0.001*** | 0.191 | 0.19 |
| Skull | MOTOR | -0.05 | 0.69 |  |  |  |  |
|  | L-DLPFC | -0.150 | 0.30 |  |  |  |  |
| Scalp | Vertex |  |  |  |  |  |  |
|  | L-DLPFC |  |  |  |  |  |  |

*^ * = ρ < 0.05, ** = ρ < 0.01 and *** = ρ < 0.001. WM (White Matter); GM (Grey Matter); CSF (cerebrospinal fluid); L-DLPFC (left dorsolateral prefrontal cortex).*

**Supplementary Table 7.** Correlation of Peak-EF with Different Anatomical Factors.

| Tissue Type | Age Group | Peak-EF (V/m) | | Paired T-Test  p-value |
| --- | --- | --- | --- | --- |
|  |  | Simulation Location | |  |
| Mean (SD) | | MOTOR | L-DLPFC |  |
| WM | Children | 2.12 (0.12) | 1.90 (0.16) | < 0.001*** |
|  | Adults | 1.84 (1.15) | 1.69 (0.13) | < 0.001*** |
|  | Elderlies | 1.93 (0.32) | 1.52 (0.15) | < 0.001*** |
| GM | Children | 1.97 (0.08) | 1.87 (0.09) | < 0.001*** |
|  | Adults | 1.75 (0.15) | 1.74 (0.12) | 0.44 |
|  | Elderlies | 1.68 (0.24) | 1.49 (0.13) | < 0.001*** |
| CSF | Children | 2.02 (0.06) | 1.91 (0.10) | < 0.001*** |
|  | Adults | 1.84 (0.16) | 1.80 (0.12) | 0.011* |
|  | Elderlies | 1.70 (0.26) | 1.55 (0.12) | 0.020* |
| Skull | Children | 3.74 (0.85) | 4.11 (1.44) | 0.1 |
|  | Adults | 4.80 (0.75) | 6.11 (1.46) | 0.002* |
|  | Elderlies | 4.79 (1.18) | 4.94 (1.24) | 0.61 |
| Scalp | Children | 2.57 (0.06) | 2.60 (0.06) | 0.23 |
|  | Adults | 2.51 (0.06) | 2.66 (0.12) | < 0.001*** |
|  | Elderlies | 2.54 (0.12) | 2.59 (0.10) | 0.29 |

*^ * = ρ < 0.05, ** = ρ < 0.01 and *** = ρ < 0.001. SD (Standard Deviation); Peak-EF (TMS Induced Electric Field Peak); WM (White Matter); GM (Grey Matter); CSF (cerebrospinal fluid); L-DLPFC (left dorsolateral prefrontal cortex).*

**Supplementary Table 8.** Statistical Analysis of Peak Electric Fields Induced by TMS under Different Simulation Locations.
